# Hydrogen sulfide acts as a double-edged sword in human hepatocellular carcinoma cells through EGFR/ERK/MMP-2 and PTEN/AKT signaling pathways

**DOI:** 10.1038/s41598-017-05457-z

**Published:** 2017-07-11

**Authors:** Dongdong Wu, Mengling Li, Wenke Tian, Shuaiwei Wang, Longzhen Cui, Hui Li, Huijuan Wang, Ailing Ji, Yanzhang Li

**Affiliations:** Henan University School of Medicine, Kaifeng, 475004 Henan China

## Abstract

Hydrogen sulfide (H_2_S) is involved in cancer biological processes. However, there are several controversies concerning the role of H_2_S in cancer development and progression. In this study, we found that the growth and migration of hepatocellular carcinoma (HCC) cells were enhanced by 10–100 μM NaHS and dose-dependently inhibited by 600–1000 μM NaHS. The apoptotic levels were reduced by 25–100 μM NaHS but increased by 400–1000 μM NaHS in HCC cells. After treatment with 25–50 μM NaHS, the protein levels of p-EGFR, p-ERK, MMP-2, and p-AKT were increased, whereas the levels of PTEN and the ratio of BAX/BCL-2 were down-regulated. Administration of 800–1000 μM NaHS showed opposite effects on these protein levels in HCC cells. However, H_2_S showed no effects on the growth, migration, apoptosis, and the protein levels of the EGFR/ERK/MMP-2 and PTEN/AKT signaling pathways in L02 cells. Furthermore, 25–100 μM NaHS promoted HCC tumor growth and blood vessel formation, while 800–1000 μM NaHS inhibited angiogenesis and tumor growth with no obvious systemic toxicity. These results indicate that H_2_S acts as a double-edged sword in HCC cells through EGFR/ERK/MMP-2 and PTEN/AKT signaling pathways. Novel H_2_S donors could be designed and applied for further antitumor research.

## Introduction

Hydrogen sulfide (H_2_S) is widely considered the third endogenous gaseous transmitter, accompanying carbon monoxide and nitric oxide^1^, and plays important roles in angiogenesis^2^, neuronal activity^3^, vascular relaxation^4^, glucose metabolism^5^, energy production^6^, and the inflammatory response^7^. However, abnormal H_2_S metabolism is associated with a number of diseases, including heart failure^8^, hypertension^9^, atherosclerosis^10^, asthma^11^, diabetes^12^, and neurodegenerative diseases^13^.

The enzymes for endogenous H_2_S production, cystathionine γ-lyase (CSE), cystathionine β-synthase (CBS), and 3-mercaptopyruvate sulfotransferase (3-MST), have been found in many cancers, including colon, liver, ovarian, breast, gastric, and prostate cancers^14^. However, the role of H_2_S in cancer development and progression is controversial. Several studies have shown that H_2_S endogenously stimulates angiogenesis and promotes tumor cell growth and proliferation^2, 14, 15^. In hepatoma cells, H_2_S is involved in maintaining the cell proliferation. Blocking H_2_S production resulted in suppression of hepatocellular carcinoma (HCC) growth by suppressing cell growth-related signaling and stimulating mitochondrial apoptosis^16^. However, treatment of human hepatoma HepG2 cells and colorectal carcinoma HCT116 cells with 400 μM GYY4137 (a slow-releasing H_2_S donor) showed anticancer activity partly by promoting apoptosis^17^. Therefore, we speculate that relatively low levels of exogenous H_2_S could promote HCC cell growth, whereas high concentrations of H_2_S might exhibit anticancer effects.

To test this hypothesis, we determined the effects of different concentrations of NaHS (an H_2_S donor) on the growth of human HCC cells *in vitro* and *in vivo* and clarified the associated molecular mechanisms.

## Materials and Methods

### Cell proliferation assay

Normal human liver cell line L02 and human HCC cell lines SMMC-7721 and Huh-7 were cultured in high-glucose Dulbecco’s modified Eagle’s medium containing 10% FBS, 100 units/mL penicillin, and 100 μg/mL streptomycin at 37 °C in a humidified atmosphere with 5% CO_2_. The cell viability was evaluated by the 3-(4,5)-dimethylthiahiazo (-z-y1)-3,5-di-phenytetrazoliumromide (MTT) assay. Cells were seeded into 96-well plates at a density of 5 × 10^3^ cells/well. After overnight incubation, cells were respectively treated with 0, 10, 25, 50, 100, 200, 400, 600, 800, and 1000 μM NaHS (Sigma-Aldrich, St. Louis, MO, USA) for 24 h. Six parallel wells were used for each concentration. MTT (Sigma-Aldrich, St. Louis, MO, USA) solution (20 μL of 5 mg/mL) was added to each well and incubated 4 h at 37 °C. After removing the medium, dimethyl sulfoxide (150 µL) was added to each well to solubilize the formazan crystals. The absorbance was detected at 490 nm on a microplate reader (Bio-Rad, CA, USA). The proliferation rate was expressed as a percentage of the untreated control. The 5-Ethynyl-2′-deoxyuridine (EdU) staining assay was performed using the Cell-Light EdU Apollo 567 *In Vitro* Imaging Kit (RiboBio, Guangzhou, Guangdong, China) according to the manufacturer’s instructions. The experiments were conducted in triplicate.

### Wound healing assay

At 48 h after seeding 2 × 10^5^ cells in 6-well plates, the cellular layer was scratched with a sterile micropipette tip. The migration distance was measured 24 h after NaHS (0–1000 μM) treatment using Image J software (National Institute for Health, Bethesda, MD, USA). The migration rate (MR) was calculated as MR (%) = [(A − B)/A] × 100, where A is the width at 0 h, and B is the width at 24 h.

### TdT-mediated dUTP-biotin nick end labeling (TUNEL) assay

TUNEL staining was performed using an *In Situ* Cell Death Detection Kit (Beyotime Biotechnology, Shanghai, China) according to the manufacturer’s protocol. The percentage of TUNEL-positive cells was measured using Image J software. Five random areas were selected for each sample (magnification 200x).

### Measurement of H_2_S levels

The concentrations of H_2_S in both cells and culture supernatant were determined using enzyme-linked immunosorbent assay (ELISA) kits according to the manufacturer’s instructions (LanpaiBio, Shanghai, China). Briefly, the cells and culture supernatant were collected to test the levels of H_2_S. Then, the standard controls were diluted, in which the concentration of H_2_S was 15 ng/L, 30 ng/L, 60 ng/L, 120 ng/L, and 180 ng/L, respectively. The samples were diluted and incubated 0.5 h at 37 °C. After H_2_S was bound and the plates were washed, the conjugate reagent was added to the well and incubated 0.5 h at 37 °C. After washing, the colour-developing agents were added to each well and incubated 15 min at 37 °C. The optical density of each well was measured with a microplate reader (Bio-Rad, CA, USA) at 450 nm. A standard curve was generated by plotting the logarithm of the mean absorbance for each standard versus the logarithm of the known H_2_S concentration. The value for the blank was subtracted from both the samples and the standard controls. The experiments were repeated three times.

### Western blotting

Total protein was extracted from L02, SMMC-7721, and Huh-7 cells. Western blotting was performed to detect the target proteins. The primary antibodies, including rabbit anti-human total protein kinase B (PKB/Akt), phospho (p)-Akt (Ser473), total extracellular signal-regulated protein kinases 1/2 (ERK1/2), p-ERK1/2 (Thr202/Tyr204), total epidermal growth factor receptor (EGFR), p-EGFR (Tyr1086), phosphatase and tensin homolog deleted on chromosome ten (PTEN), matrix metalloproteinase 2 (MMP-2), and beta-actin (β-actin) were purchased from Cell Signaling Technology (Danvers, MA, USA); rabbit anti-human B cell lymphoma/lewkmia-2 (Bcl-2), Bcl-2-associated X protein (BAX), CSE, CBS, and 3-MST antibodies were purchased from Santa Cruz Biotechnology Inc. (Santa Cruz, CA, USA). The secondary antibody, horseradish peroxidase-conjugated sheep anti-rabbit IgG was purchased from Cell Signaling Technology (Danvers, MA, USA). The bands were semi-quantified with Image J software.

### Animal study

Animal experiments were approved by the Committee of Medical Ethics and Welfare for Experimental Animals of Henan University School of Medicine (HUSOM2015-009) in compliance with the Experimental Animal Regulations formulated by the National Science and Technology Commission, China. Animal experiments were conducted in accordance with the committee’s approved guidelines. Animal studies were performed as previously described with minor modifications^18^. Sixty 4-week-old female BALB/C nude mice were purchased from Beijing HFK Bioscience Co., Ltd. (Certificate No. SCXK (Jing) 2014-0004, Beijing, China). SMMC-7721 and Huh-7 cells (5 × 10^6^ cells in 200 μL PBS) were implanted by subcutaneous injection into the right flanks of mice. At 24 h after inoculation, the mice were randomly divided into ten groups (n = 6 per group). NaHS (0–1000 μM) was continuously administered subcutaneously (near the implanted tumor) for 14 days. The tumor volumes were monitored daily and calculated as volume = L × W^2^/2, where L is the longest dimension parallel to the skin surface and W is the dimension perpendicular to L and parallel to the surface^19^. The mice were sacrificed at 24 h after the last administration. The tumors were excised and weighted to evaluate the inhibition rate (IR). The IR of tumor growth was calculated as IR (%) = [(A − B)/A] × 100, where A is the average tumor weight of the control group, and B is that of the treatment group^18^.

### Hematoxylin and eosin (HE) staining

A necropsy examination was performed immediately after sacrifice. Tumor samples and major organs, including heart, liver, spleen, lung, kidney, and brain, were fixed in 10% neutral buffered formalin, embedded in paraffin, sectioned at 5 μm thickness, and processed according to the HE staining protocol. The stained tissues were observed using an Olympus BX51 microscope (Olympus, Tokyo, Japan).

### Immunohistochemistry (IHC) and evaluation

CD34 is considered an ideal biomarker for vascular endothelial cells, and its immunostaining density is represented by the tumor microvessel density (MVD)^20^. To determine the tumor MVD, tumor tissue sections were stained by IHC using CD34 antibody (1:100, Santa Cruz, CA, USA). The sections were scanned under a light microscope with low-power magnification (100x) to identify areas with the angiogenic vessels. Stained vessels with a clearly defined lumen or well-defined linear vessel shape were counted in six high-power (400x) fields from the representative tumor zone, and the mean value was regarded as MVD.

### *In vivo* toxicity assessment

NaHS (0–1000 µM) was administrated subcutaneously once a day for 14 days. Mice were observed and weighed daily during the experimental period. The blood was collected from the heart before sacrifice at the end of the experiment. Total number of white blood cell (WBC) was measured using an automated analyzer (Beckman Coulter, Inc., Fullerton, CA, USA). The heart, liver, spleen, lung, kidney, and brain were removed and weighed immediately after sacrifice, and the relative organ weights (g/100 g body weight) were calculated.

### Statistical analysis

Data are presented as means ± standard error of the mean (SEM). The differences between multiple groups were analyzed by one-way analysis of variance (ANOVA) using SPSS 17.0 software, followed by Tukey’s test. A *P* value of less than 0.05 was considered to be statistically significant.

## Results

### The levels of H_2_S in human HCC cells were higher than those in L02 cells

**Figure 1:**
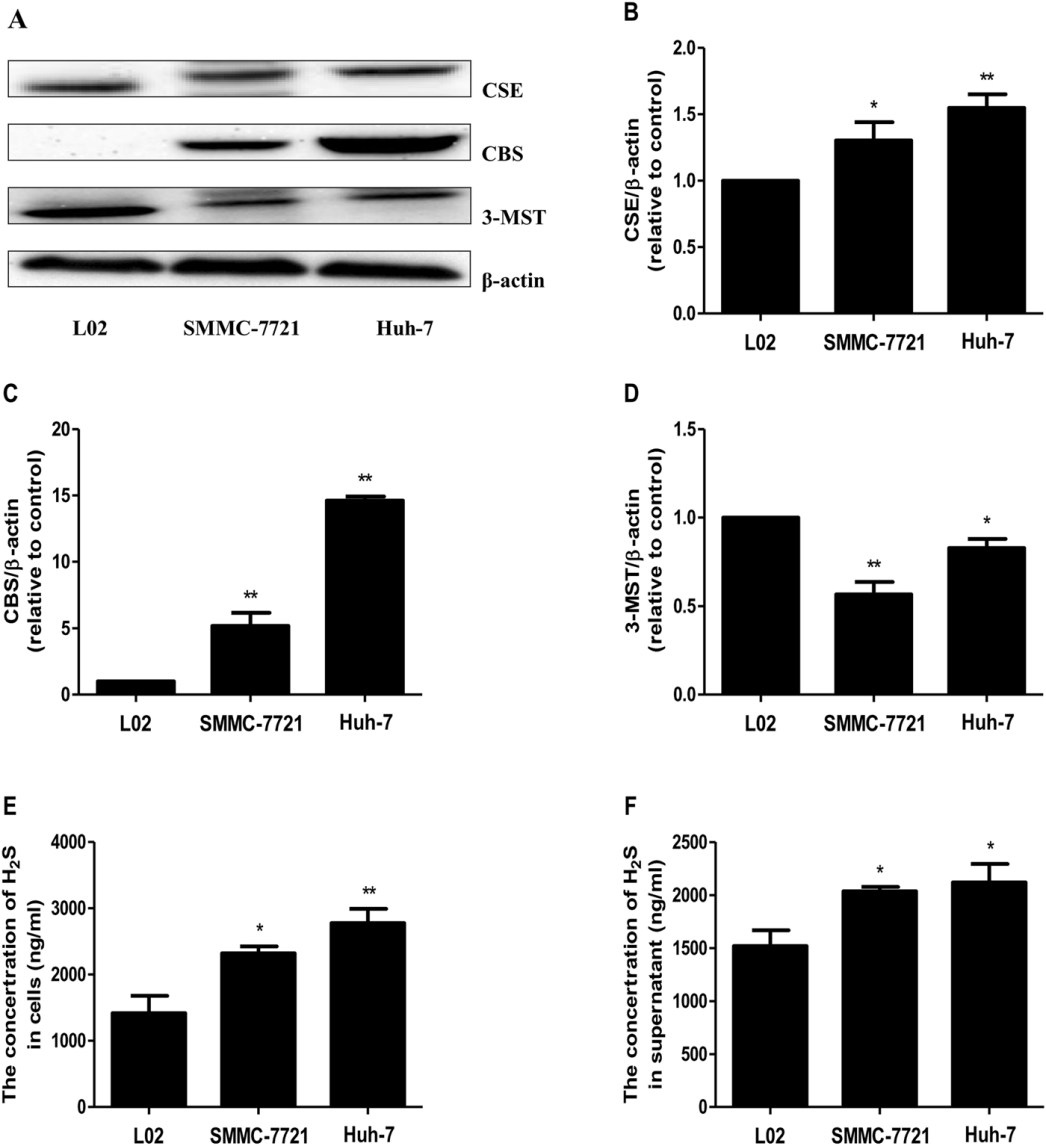
The protein expression of H_2_S-generating enzymes and the levels of H_2_S in human normal hepatocytes and HCC cells were detected. (**A**) The protein expression of CSE, CBS, and 3-MST were examined by Western blot. β-actin was used as the loading control. (**B**–**D**) Bar graphs showed the quantification of CSE, CBS, and 3-MST. The densitometry analysis of each factor was performed, normalized to the corresponding β-actin level. (**E**) The levels of H_2_S in L02 cells and human HCC cells. (**F**) The levels of H_2_S in the culture supernatant. Data are presented as mean ± SEM of three independent experiments; *P < 0.05, **P < 0.01 compared with the control group.

As shown in Fig. 1A–C, the protein levels of CSE and CBS in both SMMC-7721 and Huh-7 cells were significantly higher than those in L02 cells. While the protein levels of 3-MST in SMMC-7721 and Huh-7 cells were lower than those in L02 cells (Fig. 1A and D). Furthermore, the levels of H_2_S in SMMC-7721 and Huh-7cells, as well as in the supernatant were notably higher than those in L02 cells and the supernatant (Fig. 1E and F). These results indicated that H_2_S could be involved in the development and progression of human HCC cells.

### Dual effects of H_2_S on the growth and migration of HCC cells

**Figure 2:**
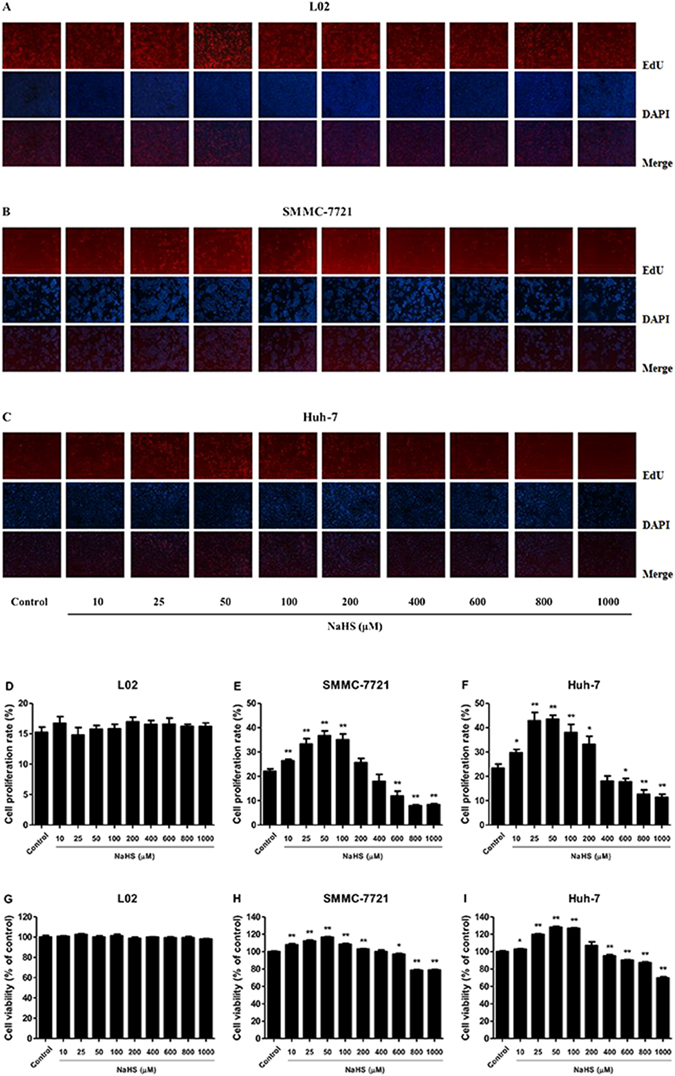
Effect of H_2_S on the proliferation and viability of human normal hepatocytes and HCC cells. (**A**–**C**) DNA replication activities of human normal hepatocytes and HCC cells in each group were examined by EdU assay; original magnification 200x. (**D**–**F**) The proliferation rate of each group was analyzed. (**G**–**I**) The percentages of viable cells were determined using MTT assay and the cell viability of every cell line without NaHS treatment was normalized as 100% and considered to be the control group. Data are presented as mean ± SEM of three independent experiments; *P < 0.05, **P < 0.01 compared with the control group.

**Figure 3:**
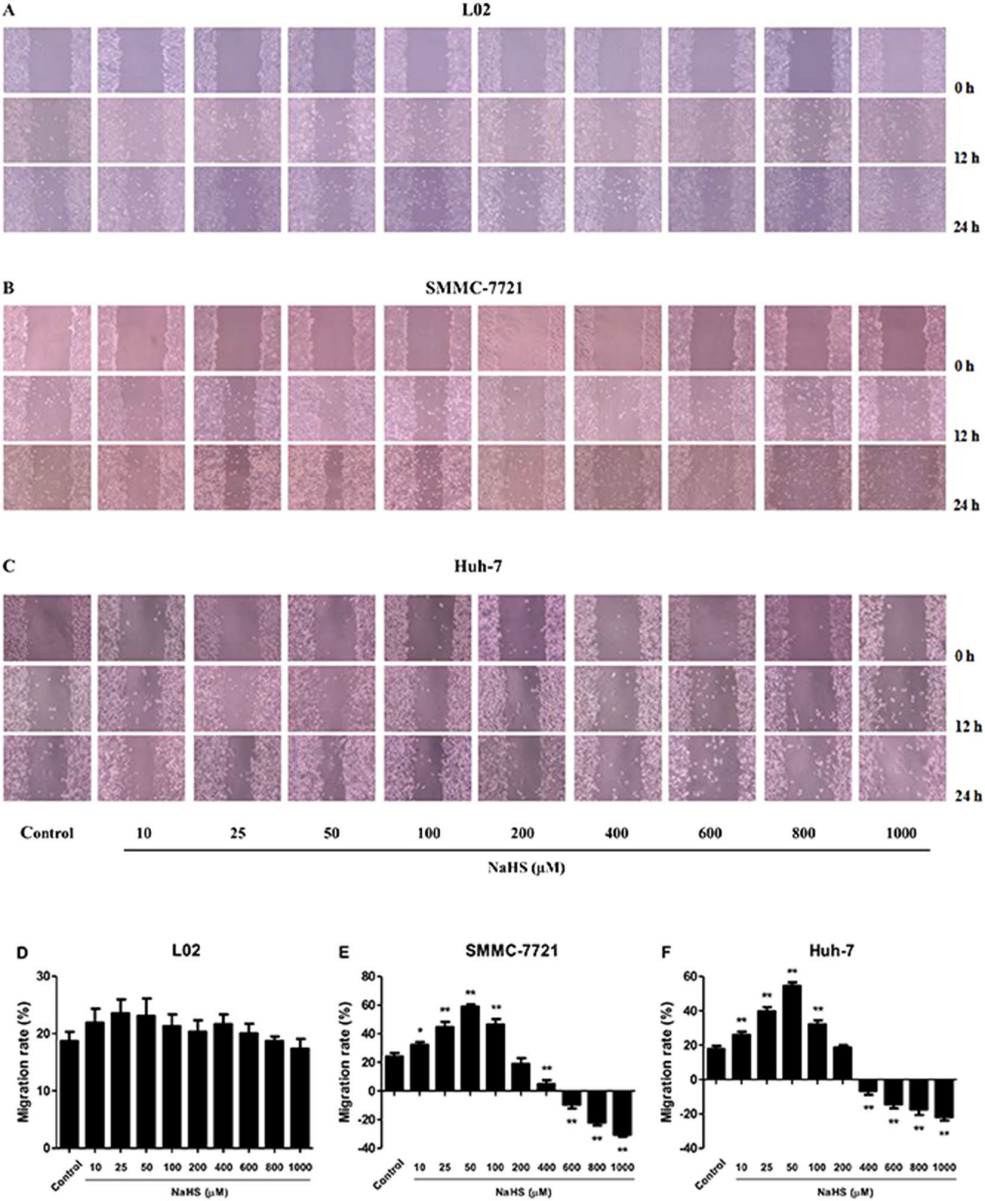
Effect of H_2_S on the mobility of human normal hepatocytes and HCC cells. (**A**–**C**) The human normal hepatocyte cell line L02 and HCC cell lines SMMC-7721 and Huh-7 were seeded into 6-well plates at 2 × 10^5^ cells/well. After incubation with NaHS at 0 (Control), 10, 25, 50, 100, 200, 400, 600, 800, and 1000 μM for 24 h, the effect of H_2_S on cell migration was measured by wound-healing assay; original magnification 40x. (**D**–**F**) The migration rates of L02, SMMC-7721, and Huh-7 cells were calculated by the formula shown above. Data are presented as mean ± SEM of three independent experiments; *P < 0.05, **P < 0.01 compared with the control group.

H_2_S had no obvious effects on the proliferation and viability of human normal hepatocytes (Fig. 2A,D and G). The proliferation and viability of SMMC-7721 and Huh-7 cells were significantly enhanced by 10–100 μM NaHS and dose-dependently inhibited by 600–1000 μM NaHS (Fig. 2B,C,E,F,H and I). H_2_S did not affect the migration of L02 cells (Fig. 3A and D). The migration distance of HCC cells was significantly increased by 10–100 μM NaHS but decreased by 400–1000 μM NaHS relative to the control group (Fig. 3B,C,E and F). These results suggested that H_2_S plays an important role in the regulation of liver cancer cell growth and migration.

### Dual effects of H_2_S on apoptosis in HCC cells

**Figure 4:**
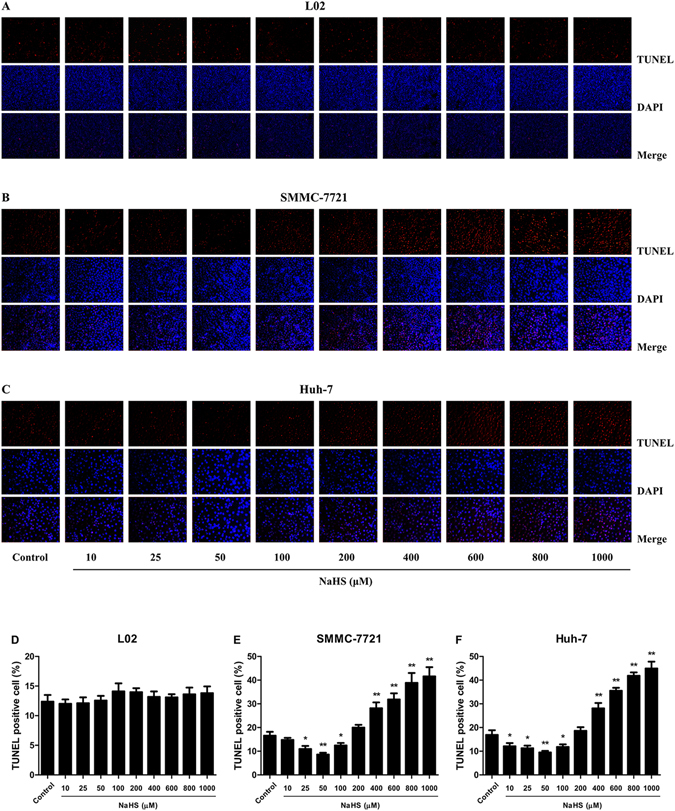
Effect of H_2_S on the apoptosis of normal human hepatocytes and HCC cells. (**A**–**C**) The human normal hepatocyte cell line L02 and HCC cell lines SMMC-7721 and Huh-7 were seeded into 6-well plates at 2 × 10^5^ cells/well. After incubation with NaHS at 0 (Control), 10, 25, 50, 100, 200, 400, 600, 800, or 1000 μM for 24 h, apoptosis was measured by TUNEL staining; original magnification 200x. (**D**–**F**) The percentages of TUNEL-positive cells were calculated. Data are presented as mean ± SEM of three independent experiments; *P < 0.05, **P < 0.01 compared with the control group.

TUNEL staining was performed to evaluate the apoptosis of L02, SMMC-7721, and Huh-7 cells with NaHS treatment. In L02 cells, there was no obvious change between each group (Fig. 4A and D). The numbers of apoptotic cells were significantly reduced by 25–100 μM NaHS but dose-dependently increased by 400–1000 μM NaHS in both SMMC-7721 and Huh-7 cells (Fig. 4B,C,E and F).

### H_2_S mediated the EGFR/ERK/MMP-2 pathway in HCC cells

**Figure 5:**
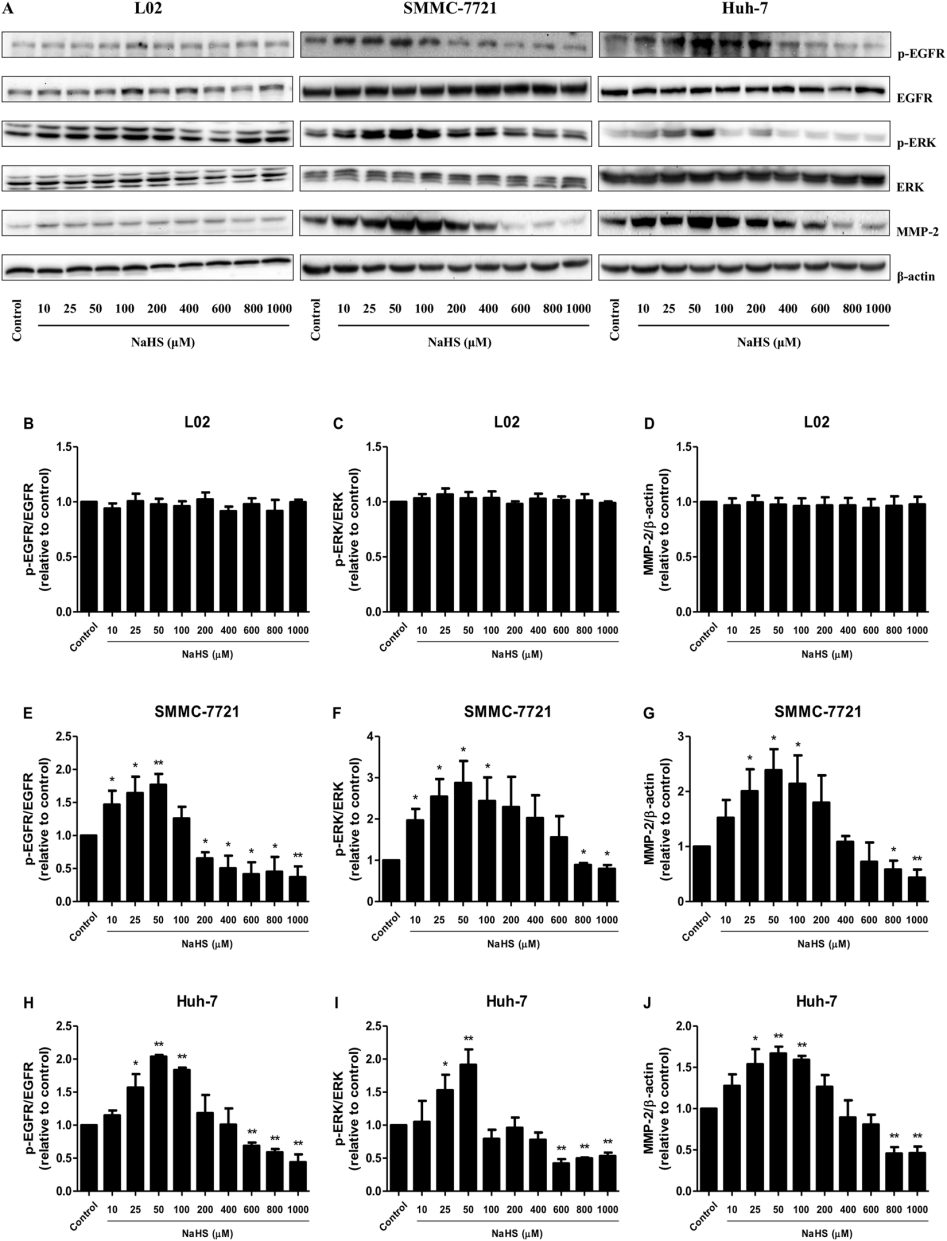
H_2_S mediated the EGFR/ERK/MMP-2 pathway in human HCC cells. L02, SMMC-7721, and Huh-7 cells were cultured in 6-well plates and treated with NaHS at concentrations of 0 (Control), 10, 25, 50, 100, 200, 400, 600, 800, and 1000 μM for 24 h, then the protein expression levels of the indicated factors were examined by Western blot. β-actin was used as the loading control. The densitometry analysis of each factor was performed, normalized to the corresponding β-actin level. Values were presented as mean ± SEM of three independent experiments; *P < 0.05, **P < 0.01 compared with the control group.

As shown in Fig. 5A–D, H_2_S showed no obvious effect on the protein expression of each group in L02 cells. EGFR phosphorylation results in ERK-mediated signaling transduction, which supports cell growth and facilitates cellular motility^21, 22^. Treatment with 25–50 μM NaHS dose-dependently increased EGFR and ERK phosphorylation in HCC cells with no obvious changes in total expression. However, 800–1000 μM NaHS reduced EGFR and ERK phosphorylation levels (Fig. 5A,E,F,H and I). Because MMP-2 is involved in cancer cell migration and invasion, which could be regulated by ERK1/2 signaling^23, 24^, MMP-2 expression in HCC cells was further examined. In HCC cells, 25–100 μM NaHS significantly increased MMP-2 protein expression, whereas 800–1000 μM NaHS decreased expression (Fig. 5A,G and J).

### H_2_S mediated the PTEN/AKT pathway in HCC cells

**Figure 6:**
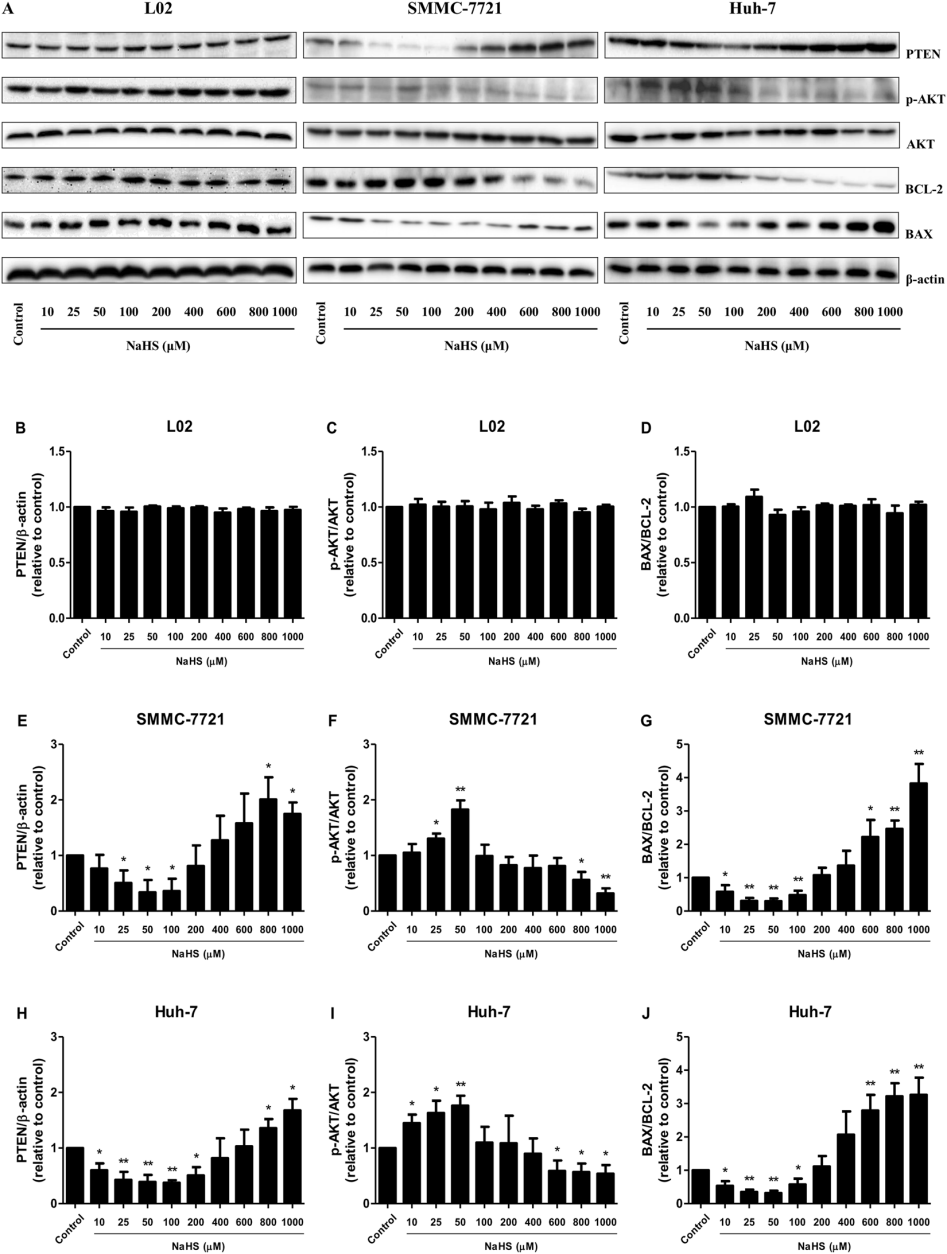
H_2_S mediated the PTEN/AKT pathway in human HCC cells. L02, SMMC-7721, and Huh-7 cells were cultured in 6-well plates and treated with NaHS at concentrations of 0 (Control), 10, 25, 50, 100, 200, 400, 600, 800, and 1000 μM for 24 h, then the protein expression levels of the indicated factors were examined by Western blot. β-actin was used as the loading control. The densitometry analysis of each factor was performed, normalized to the corresponding β-actin level. Values were presented as mean ± SEM of three independent experiments; *P < 0.05, **P < 0.01 compared with the control group.

There was no change of the protein level of each group in L02 cells (Fig. 6A–D). Increasing evidence indicates that AKT activation is positively correlated with cancer progression^24, 25^. We found that 25–50 μM NaHS dose-dependently up-regulated p-AKT without changing the total AKT level in HCC cells. However, 800–1000 μM NaHS reduced AKT phosphorylation (Fig. 6A,F and I). PTEN is a key negative regulator of the PI3K/AKT pathway and is frequently inactivated in human tumors^26^. Treatment with 25–100 μM NaHS reduced PTEN expression, whereas 800–1000 μM NaHS increased the level of PTEN protein (Fig. 5A,E and H). The ratio between BAX and BCL-2 proteins is an important factor in apoptosis regulation^27, 28^. An increased BAX/BCL-2 ratio is sufficient to directly activate mitochondrial apoptosis in mammalian cells^27, 29^. Treatment with 10–100 μM NaHS decreased the BAX/BCL-2 ratio, whereas 600–1000 μM NaHS increased it in HCC cells (Fig. 6A,G and J).

### H_2_S modulated the growth and angiogenesis of HCC xenograft tumors in nude mice

**Figure 7:**
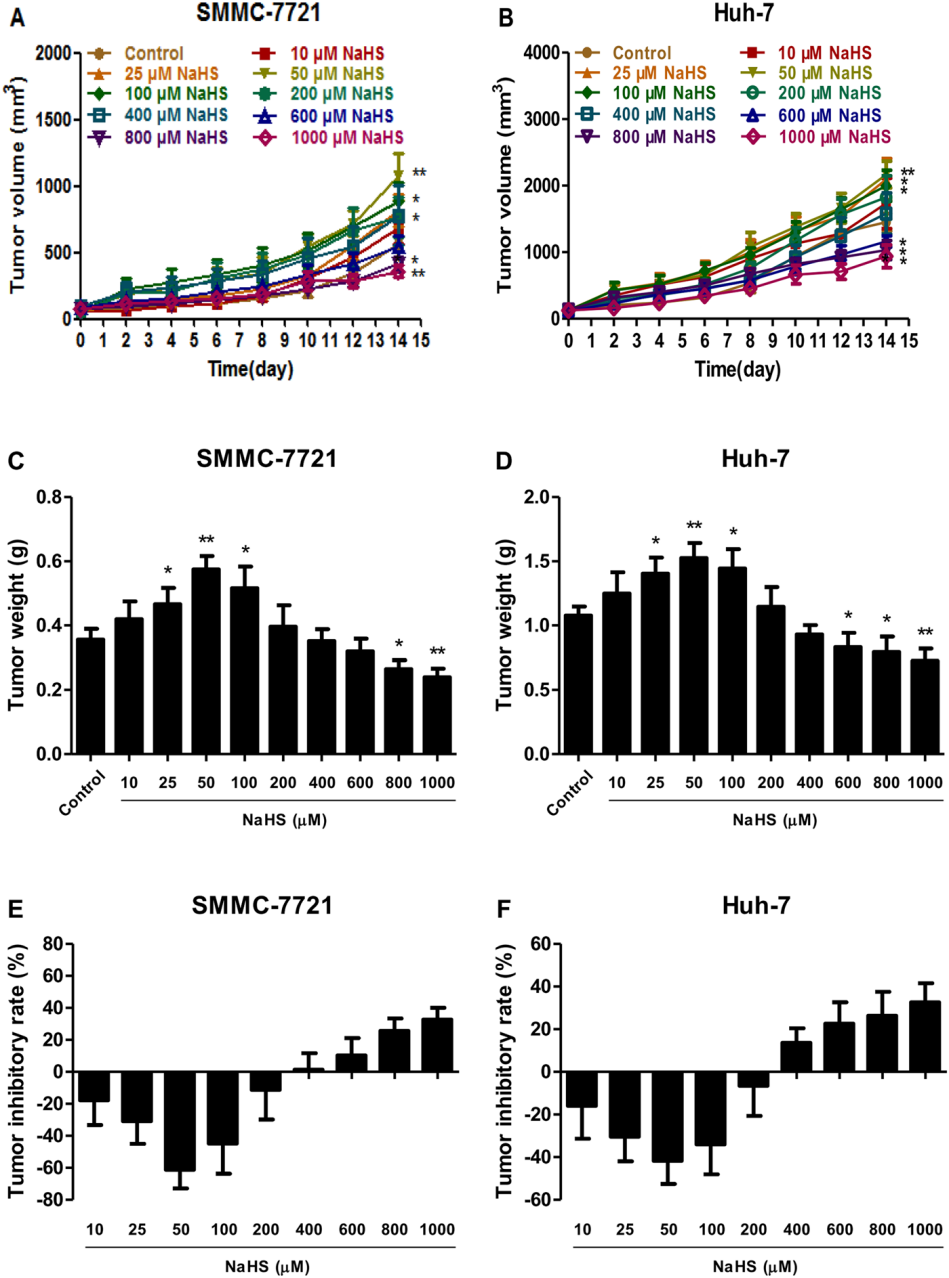
Effect of H_2_S on the growth of SMMC-7721 and Huh-7 xenograft tumors in nude mice. (**A** and **B**) The tumor volume was measured every day and calculated according to the equation: tumor volume = length × width^2^/2. (**C** and **D**) At the end of the experiment (day 14), the tumors were harvested and weighed. (**E** and **F**) The tumor inhibitory rate was calculated. Values are presented as mean ± SEM (n = 6); *P < 0.05, **P < 0.01 compared with the control group.

**Figure 8:**
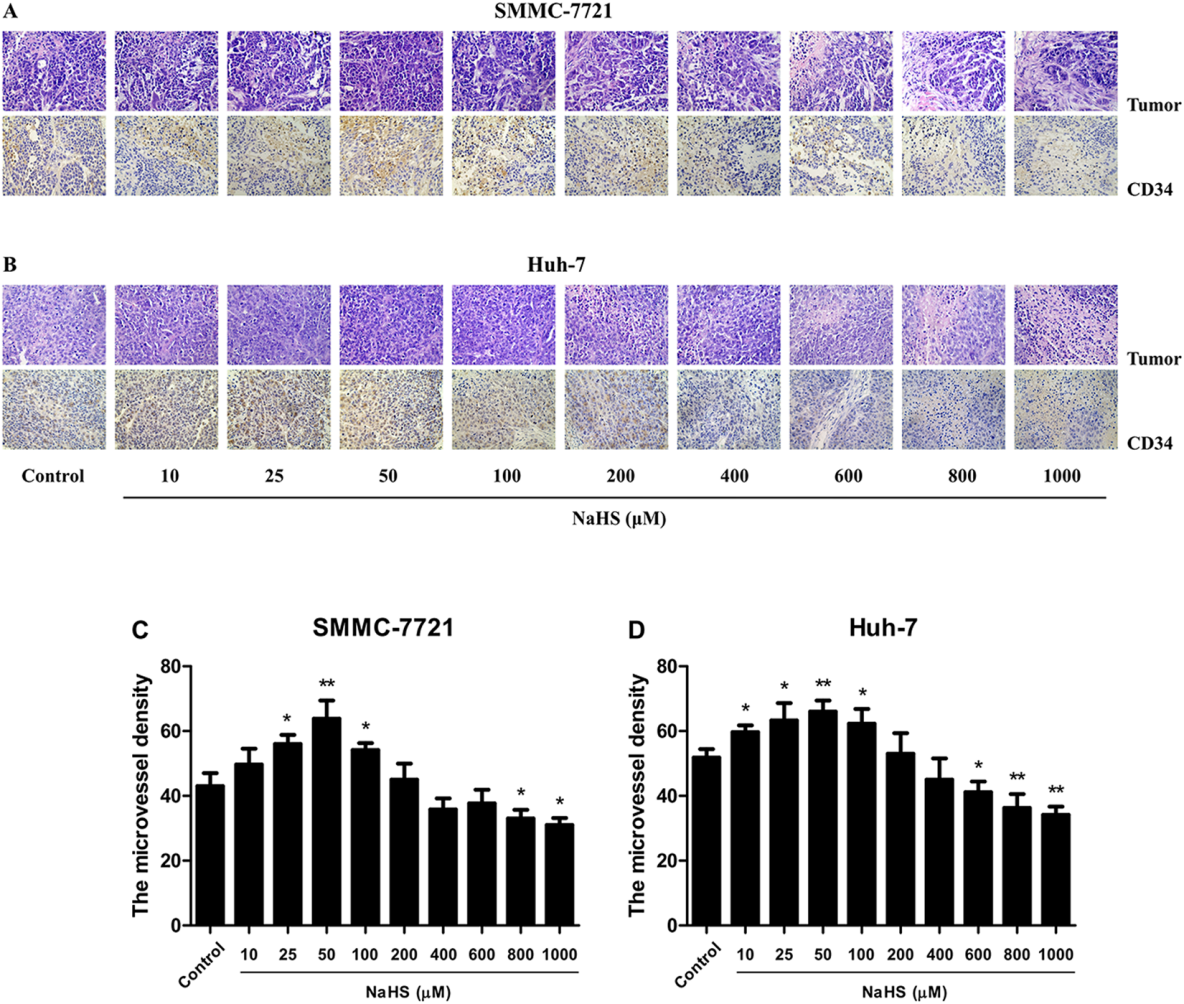
Effect of H_2_S on the angiogenesis of HCC xenografts. (**A** and **B**) Tumors were harvested and used for HE and CD34 staining; original magnification 400x. (**C** and **D**) MVD was determined by counting the number of microvessels in HCC xenografts. Values are presented as mean ± SEM (n = 6); *P < 0.05, **P < 0.01 compared with the control group.

SMMC-7721 and Huh-7 cells have been widely used to establish xenograft tumor models^30–32^. We therefore investigated the effect of H_2_S on HCC xenograft growth in BALB/c nude mice. Treatment with 25–100 μM NaHS promoted the growth of HCC xenograft tumors in nude mice; however, 800–1000 μM NaHS exhibited significant anti-HCC effects (Fig. 7A–F). Treatment with 25–100 μM NaHS significantly increased the MVD, whereas 800–1000 μM NaHS dose-dependently decreased the MVD (Fig. 8A–D).

### H_2_S showed no obvious toxicity in nude mice bearing HCC xenografts

**Figure 9:**
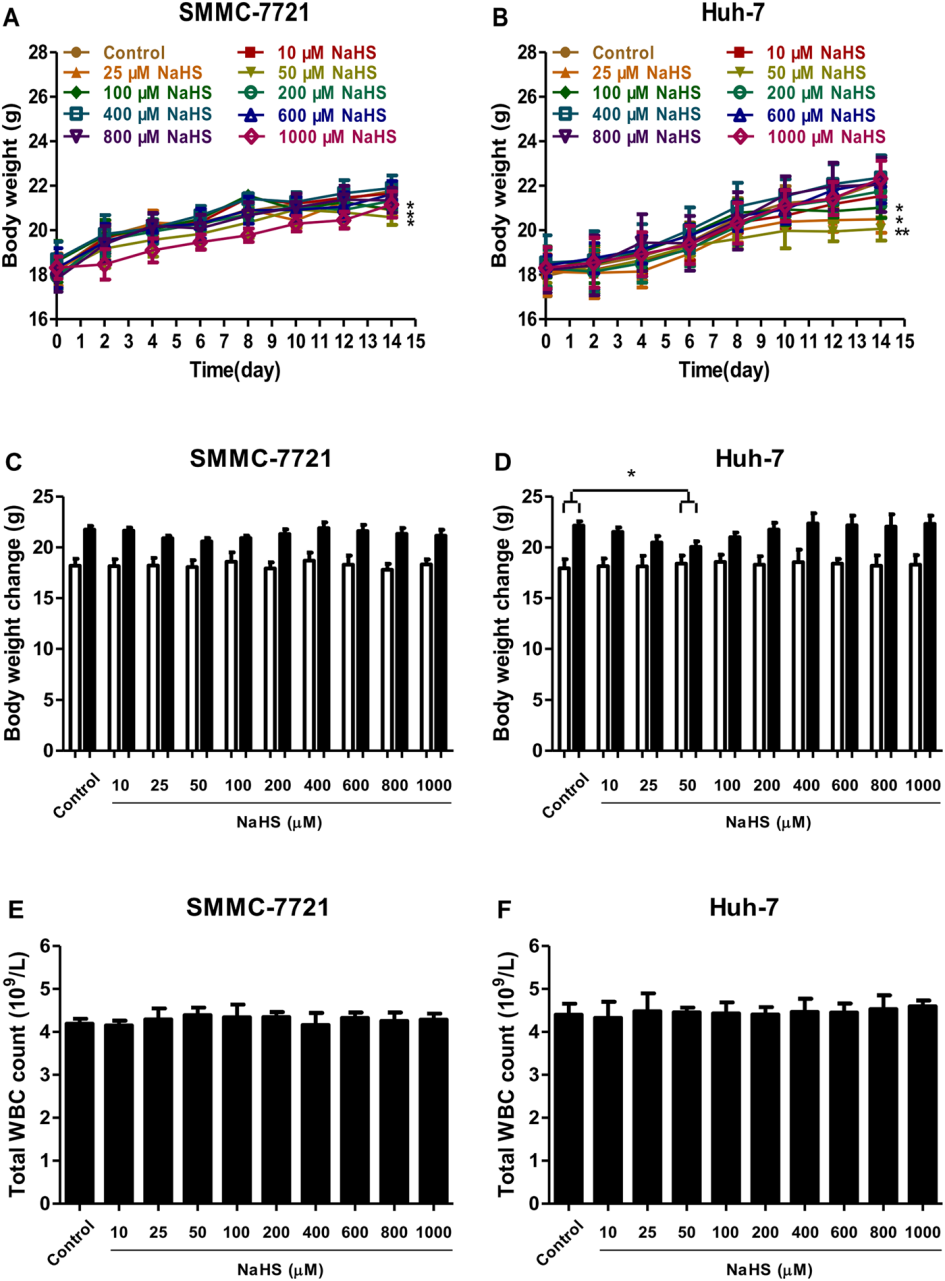
Body weight change and total WBC count of BALB/c nude mice after subcutaneous administration of NaHS at the indicated concentrations for 14 days. (**A** and **B**) The body weight change curve of each group during the experiment. (**C** and **D**) The body weight of each group on the first day (day 0) and the last day (day 14). (**E** and **F**) The total WBC count of each group was examined at the end of the experiment. Values are presented as mean ± SEM (n = 6); *P < 0.05, **P < 0.01 compared with the control group.

**Figure 10:**
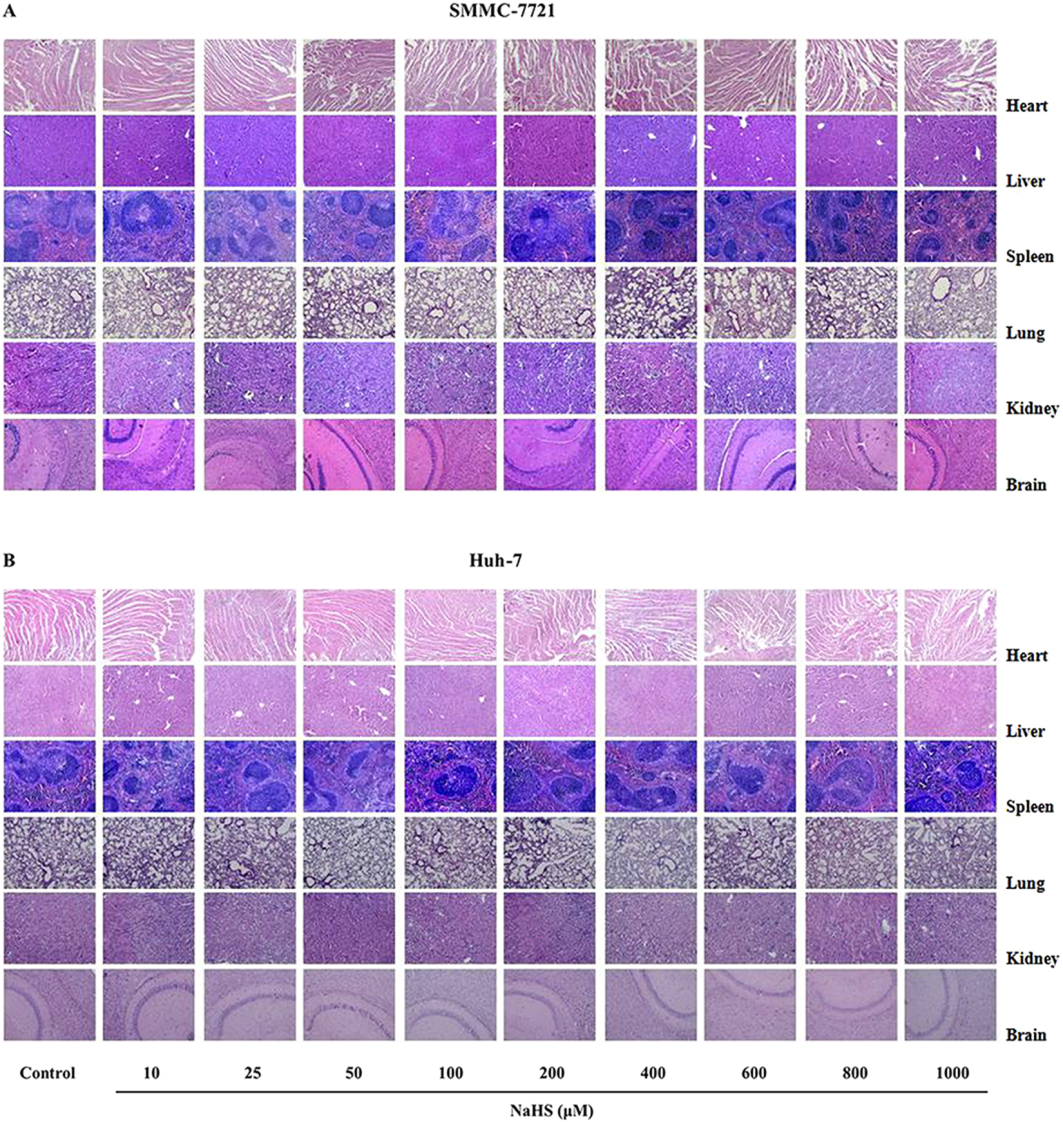
Effect of H_2_S on the structures of the heart, liver, spleen, lung, kidney, and brain in mice. Histopathology of the above-mentioned organs of the mice bearing SMMC-7721 (**A**) and Huh-7 (**B**) xenografts after treatment with NaHS at the indicated concentrations for 14 days. No significant morphological damage was observed. HE staining; original magnification 100x.

The body weights of mice were monitored every day throughout the experiment. Initially, there were no significant differences between the groups. However, at the end of the study, mice administered 25–100 μM NaHS had significantly lower body weights than the control group (Fig. 9A and B). Since loss of body weight is a common feature of cancer development^33^, 25–100 μM NaHS might significantly accelerate cancer progression. Although the body weights in each group increased slowly during the experiment, the increase for mice administered 50 μM NaHS was the lowest (Fig. 9C and D). There were no significant differences in the total WBC count (Fig. 9E and F) and relative weights of heart, liver, spleen, lung, kidney, and brain (Tables 1 and 2). Furthermore, there were no obvious morphological differences of heart, liver, spleen, lung, kidney, and brain in each group (Fig. 10A and B).

**Table 1:** Relative organ weight of nude mice bearing SMMC-7721 xenograft after the subcutaneous administration of NaHS for 14 days.

NaHS (μM)	Heart	Liver	Spleen	Lung	Kidney	Brain
Control (0)	0.53 ± 0.03	5.99 ± 0.17	0.58 ± 0.04	0.83 ± 0.03	1.91 ± 0.17	1.88 ± 0.02
10	0.59 ± 0.02	6.02 ± 0.21	0.63 ± 0.04	0.88 ± 0.09	1.96 ± 0.15	1.91 ± 0.03
25	0.61 ± 0.04	6.32 ± 0.13	0.62 ± 0.02	0.91 ± 0.11	1.98 ± 0.10	1.98 ± 0.05
50	0.62 ± 0.02	6.70 ± 0.41	0.60 ± 0.03	0.80 ± 0.05	1.99 ± 0.14	2.02 ± 0.04
100	0.63 ± 0.03	6.42 ± 0.25	0.61 ± 0.04	0.93 ± 0.15	1.97 ± 0.17	1.92 ± 0.07
200	0.53 ± 0.02	6.35 ± 0.22	0.62 ± 0.02	0.77 ± 0.03	1.98 ± 0.14	1.89 ± 0.06
400	0.56 ± 0.03	6.34 ± 0.16	0.61 ± 0.03	0.75 ± 0.10	1.97 ± 0.11	1.91 ± 0.08
600	0.60 ± 0.07	6.15 ± 0.22	0.56 ± 0.02	0.76 ± 0.08	2.02 ± 0.14	1.88 ± 0.06
800	0.60 ± 0.03	6.24 ± 0.15	0.64 ± 0.06	0.80 ± 0.09	2.03 ± 0.15	1.93 ± 0.10
1000	0.54 ± 0.04	6.08 ± 0.18	0.55 ± 0.02	0.90 ± 0.13	2.04 ± 0.13	1.90 ± 0.07

**Table 2:** Relative organ weight of nude mice bearing Huh-7 xenograft after the subcutaneous administration of NaHS for 14 days.

NaHS (μM)	Heart	Liver	Spleen	Lung	Kidney	Brain
Control (0)	0.61 ± 0.03	5.96 ± 0.18	0.56 ± 0.02	0.74 ± 0.04	1.89 ± 0.03	1.95 ± 0.04
10	0.58 ± 0.02	5.87 ± 0.14	0.53 ± 0.04	0.79 ± 0.05	1.87 ± 0.05	1.91 ± 0.05
25	0.61 ± 0.02	5.86 ± 0.24	0.61 ± 0.04	0.88 ± 0.04	1.95 ± 0.07	1.91 ± 0.03
50	0.65 ± 0.04	6.13 ± 0.29	0.65 ± 0.02	0.84 ± 0.04	1.93 ± 0.03	2.03 ± 0.03
100	0.66 ± 0.02	6.04 ± 0.21	0.56 ± 0.03	0.86 ± 0.05	1.90 ± 0.06	2.01 ± 0.02
200	0.57 ± 0.02	5.84 ± 0.15	0.53 ± 0.04	0.80 ± 0.03	1.91 ± 0.08	1.93 ± 0.04
400	0.63 ± 0.05	5.79 ± 0.26	0.60 ± 0.04	0.85 ± 0.09	2.02 ± 0.09	1.91 ± 0.07
600	0.59 ± 0.04	6.17 ± 0.21	0.57 ± 0.06	0.86 ± 0.10	1.89 ± 0.06	1.93 ± 0.04
800	0.61 ± 0.05	6.02 ± 0.22	0.58 ± 0.04	0.82 ± 0.04	1.91 ± 0.07	1.97 ± 0.06
1000	0.62 ± 0.04	5.91 ± 0.15	0.56 ± 0.05	0.86 ± 0.03	1.95 ± 0.09	1.92 ± 0.05

## Discussion

Increasing evidence suggests that H_2_S plays important roles in many physiological and pathophysiological processes in mammals^34^. However, the role of H_2_S in cancer development and progression is controversial. We proposed that treatment with relatively low levels of exogenous H_2_S could promote cancer cell growth, whereas relatively high concentrations of H_2_S might exhibit anticancer effects^14^. To test our hypothesis, normal human liver cell line L02 and human HCC cells were used to evaluate the effects of H_2_S *in vitro* and *in vivo*. The results showed that the levels of H_2_S in human HCC cells and the supernatant were significantly higher than those in L02 cells and the supernatant, suggesting that H_2_S is involved in the development and progression of human HCC cells. In addition, we found that H_2_S showed no obvious effects on the proliferation, viability, and migration of human normal hepatocytes. Treatment with 10–100 μM NaHS significantly promoted the growth and migration of HCC cells, whereas 600–1000 μM NaHS exhibited opposite effects in a dose-dependent manner. These results showed that H_2_S could play an important role in the proliferation, viability, and migration of human HCC cells.

To elucidate the molecular mechanisms by which H_2_S affects the biological behaviors of HCC cells, we measured the expression levels of several growth factors and proteins in HCC cells. EGFR, a member of the receptor tyrosine kinase family, plays a pivotal role in several cellular functions and is considered an attractive target for cancer therapy^24^. EGFR activation is involved in cancer cell proliferation, migration, and invasion, and it is overexpressed in various cancers including HCC^35, 36^. EGFR phosphorylation can activate several downstream signaling pathways including PI3K/AKT and MAPK/MEK/ERK^16, 35^. MMP-2 is positively regulated by the ERK pathway and regulates degradation of extracellular matrix components, which plays a key role in cancer metastasis^24, 37^. A recent study found that 500 μM NaHS markedly increased MMP-2 expression in human PLC/PRF/5 hepatoma cells^38^. Our results herein demonstrated that 25–50 μM NaHS increased EGFR and ERK1/2 phosphorylation, whereas they were decreased by 800–1000 μM NaHS. Furthermore, 25–100 μM NaHS significantly increased MMP-2 protein expression, whereas 800–1000 μM NaHS decreased its expression in HCC cells. The inconsistent results may be attributable to the different physiological properties among HCC cell lines. Furthermore, H_2_S showed no obvious effect on the protein expression of each group in L02 cells. These results suggested that H_2_S regulates the growth and migration of HCC cells through the EGFR/ERK/MMP-2 signaling pathway.

Apoptosis, an intrinsic cell-suicide program, is critical for normal development and maintenance of tissue homeostasis in multicellular organisms^14, 39^. Herein, we examined apoptosis and the expression levels of major apoptosis-related proteins. Our results demonstrated that 25–100 μM NaHS significantly reduced apoptosis; however, 400–1000 μM NaHS dose-dependently increased apoptosis in HCC cells. There was no obvious change between each group in L02 cells. In addition, treatment with 400–1000 μM NaHS decreased the migration of HCC cells, suggesting that high concentrations of NaHS could induce apoptosis. The PI3K/AKT pathway plays a critical role in the proliferation, migration, metabolism, and apoptosis of cancer cells^23, 35, 40^. PTEN, a tumor suppressor protein, possesses alkaline phosphatase and protein phosphatase activities and can block PI3K/AKT signaling^26, 41^. The decreased expression of PTEN may result in the activation of the AKT/ERK pathways, leading to the proliferation and migration of HCC cells. However, the increased level of PTEN could induce apoptosis of HCC cells. We examined the p-AKT and PTEN expression levels in HCC cells after treatment with H_2_S and found that 25–50 μM NaHS up-regulated AKT phosphorylation, and 25–100 µM NaHS decreased PTEN expression; however, 800–1000 μM NaHS dose-dependently reduced AKT phosphorylation and increased PTEN expression. BAX and BCL-2 are the downstream apoptosis regulators of the PI3K/AKT pathway, and the BAX/BCL-2 ratio is widely used as an important factor in the regulation of apoptosis^29, 30^. We found that 10–100 μM NaHS decreased the BAX/BCL-2 ratio, whereas 600–1000 μM NaHS dose-dependently increased the BAX/BCL-2 ratio in HCC cells. However, there was no obvious change of the protein level of each group in L02 cells. These results together indicated that PTEN/PI3K/AKT signaling might represent another mechanism underlying the effects of H_2_S on HCC cell growth and migration.

Consistent with the *in vitro* findings, 25–100 μM NaHS significantly promoted xenograft tumor growth in nude mice; however, 800–1000 μM NaHS exhibited dose-dependent anti-HCC effects. Angiogenesis plays a key role in solid tumor growth, invasion, and metastasis and is an attractive target for tumor therapy^14, 18, 39^. To determine the effect of H_2_S on angiogenesis, we observed the MVD in dissected tumor tissues by anti-CD34 immunostaining and found that it was significantly increased with 25–100 μM NaHS treatment, suggesting that H_2_S had a pro-angiogenic effect in agreement with previous findings^15^. Intriguingly, 800–1000 μM NaHS dose-dependently reduced the MVD, consistent with the effect of H_2_S on HCC xenograft tumor growth in nude mice. These results demonstrated that H_2_S could regulate tumor growth by mediating angiogenesis. Furthermore, there were no significant differences in the relative organ weights and morphologies of heart, liver, spleen, lung, kidney, and brain or in total WBC count among the groups, indicating no obvious systemic toxicity. Thus, treatment with relatively high concentrations of an H_2_S donor may effectively exert antitumor effects.

In conclusion, our data indicate that the effect of H_2_S on HCC cells is a double-edged sword mediated by EGFR/ERK/MMP-2 and PTEN/AKT signaling pathways. Novel slow-releasing H_2_S donors and H_2_S-releasing hybrid drugs could be designed and applied for further antitumor research.
